# Comparative Study on Nitrogen and Phosphorus Removal Efficiency and Rhizosphere Microbial Mechanisms of Six Wetland Plants in Eutrophic Water

**DOI:** 10.3390/plants15091346

**Published:** 2026-04-28

**Authors:** Haoliang Cheng, Jingjing He, Xuan Zhang, Yongwen Huang, Wen Jiang

**Affiliations:** 1 School of Civil Engineering, Southwest Forestry University, Kunming 650224, China; 15619059276@163.com (J.H.);; 2 Faculty of Civil Engineering and Mechanics, Kunming University of Science and Technology, Kunming 650500, China

**Keywords:** wetland plants, nitrogen and phosphorus removal, rhizosphere microorganisms, plant–microbe interactions

## Abstract

To address the limited understanding of interspecific differences in eutrophic-water remediation, six representative wetland plants—*Myriophyllum spicatum*, *Oenanthe javanica*, *Zizania latifolia*, *Ipomoea aquatica*, *Iris pseudacorus*, and *Typha orientalis*—were evaluated in a unified hydroponic system. The removal efficiencies of total nitrogen (TN), total phosphorus (TP), and ammonium nitrogen (NH_4_^+^-N) were compared together with plant biomass accumulation and root-associated and fepiphytic microbial community characteristics. The results showed marked interspecific differences in growth and pollutant removal, with the *M. spicatum* treatment exhibiting the highest overall purification performance, achieving removal rates of 83.3% for NH_4_^+^-N, 87.3% for TN, and 78.6% for TP after 42 days. Community-composition analysis suggested that the superior purification performance of *M. spicatum* was associated with a greater relative abundance of Proteobacteria and putative nitrogen- and phosphorus-cycling bacterial groups. By integrating a plant-free control with a side-by-side comparison of six wetland plants under identical hydroponic conditions, this study establishes a comparative framework linking nutrient removal to plant growth and microbial community assembly. Overall, *M. spicatum* was identified as the most promising species, providing new insight for wetland-plant selection and eutrophic-water remediation.

## 1. Introduction

Water eutrophication, characterized by cyanobacterial blooms, water quality deterioration, and degradation of aquatic ecosystem structure, has become one of the major environmental challenges in global water management [1]. Excessive inputs of nitrogen (N) and phosphorus (P) disrupt nutrient balance in aquatic ecosystems and alter microbial community structure and biogeochemical cycling processes, thereby reducing ecosystem stability and ecological service functions [2]. Although physical and chemical treatments can rapidly decrease nutrient concentrations, their high energy consumption, operational costs, and potential risks of secondary pollution restrict their long-term application in large-scale water restoration projects [3,4,5,6].

Plant-based ecological remediation has therefore emerged as a sustainable and environmentally friendly strategy for controlling eutrophic water pollution [7,8,9]. Wetland plants can directly remove inorganic nitrogen and phosphorus through uptake and assimilation, while also regulating rhizosphere or epiphytic microbial communities that facilitate nitrogen transformation and phosphorus immobilization processes [10,11,12]. Through these plant–microbe interactions, nutrient removal is achieved via coupled biological processes involving plant assimilation and microbial metabolism.

Previous studies have demonstrated that different functional types of aquatic plants exhibit considerable variation in nutrient removal efficiency and their underlying mechanisms [13,14,15]. However, most existing research focuses on single plant species or individual pollution indicators, and systematic comparisons among multiple plant species under identical environmental conditions remain limited [16,17,18,19]. In particular, the differences in plant–microbe synergistic mechanisms among plant types have not been comprehensively elucidated. The competitive performance between submerged and emergent plants under high nutrient loading conditions, as well as the associated evolution of functional microbial networks, remains insufficiently understood [20,21,22]. Variations in root morphology, oxygen release capacity, biomass accumulation, and carbon exudation may shape rhizosphere microbial communities and thereby influence nutrient removal efficiency and system stability [23,24,25].

Submerged plants such as *Myriophyllum* spp. have demonstrated strong potential for eutrophic water remediation because of their large surface area for nutrient exchange and direct contact with the water column [26,27]. Emergent plants, including *Typha* spp., *Iris pseudacorus*, *Oenanthe javanica*, *Zizania latifolia*, and *Ipomoea aquatica*, are widely used in constructed wetlands and ecological floating island systems due to their high adaptability and ease of management [28,29,30,31]. However, systematic comparisons among these plant types in terms of nutrient removal performance and plant–microbe synergistic mechanisms remain limited [32,33,34].

To address this gap and to provide a practical basis for plant selection and configuration in eutrophic water remediation, Hongqi River—an inflow river of Qilu Lake characterized by high nitrogen and phosphorus levels and classified as inferior Class V water—was selected as the target water source. A unified hydroponic system was established, and a wetland plant configuration strategy integrating pollution reduction with resource utilization was proposed. Six representative wetland plant species, including vegetable-type aquatic plants (*O. javanica*, *Z. latifolia*, and *I. aquatica*) and ornamental plants (*M. spicatum*, *T. orientalis*, and *I. pseudacorus*), were selected for comparative experiments. The removal efficiencies of total nitrogen (TN), total phosphorus (TP), and ammonium nitrogen (NH_4_^+^-N) were evaluated, and plant biomass dynamics together with rhizosphere microbial community structures were analyzed to investigate differences in nitrogen and phosphorus uptake and transformation among plant types, as well as their plant–microbe interactions. This study aims to provide a scientific basis for optimizing plant configuration in pilot-scale wetland systems and to support the synergistic improvement of ecological functions and resource utilization.

## 2. Materials and Methods

### 2.1. Experimental Materials

The experimental plants were obtained from a local nursery base in Tonghai County, Yunnan Province, China. Both submerged and emergent functional plant types were included, namely *Myriophyllum spicatum*, *Oenanthe javanica* (“Daye Shuiqin”), *Zizania latifolia* (“Dianjiao No. 1”), *Ipomoea aquatica* (“Liuyekongxincai”), *Iris pseudacorus*, and *Typha orientalis*. All plants were artificially propagated individuals with consistent sources and good growth conditions. The experiment was initiated on 13 September 2025.

Prior to the experiment, all plants were uniformly screened. Individuals showing healthy growth, free from pests and diseases, and with relatively similar sizes were selected for the experiment to reduce the influence of individual differences on the results. The initial morphological characteristics of the plants were as follows: plant height of *Z*. *latifolia* was 25.6 ± 1.2 cm with a stem diameter of 0.8 ± 0.1 cm; plant height of *O*. *javanica* was 20.1 ± 0.8 cm with a stem diameter of 0.3 ± 0.05 cm; plant height of *I*. *aquatica* was 18.5 ± 0.6 cm with a stem diameter of 0.2 ± 0.03 cm; plant height of *T*. *orientalis* was 30.4 ± 2.1 cm; plant height of *I*. *pseudacorus* was 28.7 ± 1.9 cm; and the plant length of *M*. *spicatum* was 22.3 ± 1.5 cm.

Before entering the hydroponic system, the roots or attached plant surfaces were washed with deionized water three times (5 min each) to remove surface sediments and loosely attached impurities, thereby reducing the interference of external materials on water quality changes and microbial analysis. After the treatment, the plants were immediately used for the hydroponic experiment.

### 2.2. Raw Water Quality

The experimental water was collected from the Hongqi River (102°41′ E, 24°05′ N), a tributary inflowing into Qilu Lake. The main water quality parameters of the raw water are shown in Table 1. At the time of sampling, the initial concentrations of total nitrogen (TN), total phosphorus (TP), and ammonium nitrogen (NH_4_^+^-N) were 16.6 mg L^−1^, 0.56 mg L^−1^, and 1.92 mg L^−1^, respectively, indicating eutrophic conditions (TN > 2 mg L^−1^, TP > 0.2 mg L^−1^).

The pH of the raw water was 7.8 ± 0.2, indicating neutral to slightly alkaline conditions, which were suitable for the growth and enzymatic reactions of functional microorganisms involved in nitrogen and phosphorus cycling, such as nitrifying and denitrifying bacteria. The dissolved oxygen (DO) concentration was 5.5 ± 0.3 mg L^−1^, representing aerobic conditions that provided a basic environmental condition for aerobic degradation processes in the plant rhizosphere. At the same time, this DO level could create microenvironments suitable for facultative anaerobic microorganisms involved in denitrification, which is consistent with the water environmental characteristics in the operation of constructed wetlands.

To remove suspended particles from the water, the collected raw water was filtered through a 0.45 μm membrane filter before the experiment to ensure that nutrients mainly existed in dissolved forms during subsequent analyses.

### 2.3. Experimental Setup and Operating Conditions

#### 2.3.1. Experimental Equipment and Reagents

Water quality analysis was performed using an LH-BW5 water quality analyzer (Lohand Biological, Hangzhou, China; precision ± 0.01 mg L^−1^). The cultivation environment was controlled using an RXZ-500D artificial climate chamber (Ningbo Jiangnan, Ningbo, China; temperature control accuracy ± 0.5 °C). Plant biomass was measured using an FA2004 electronic analytical balance (Shanghai Precision, Shanghai, China; precision 0.0001 g). Rhizosphere microbial sequencing was conducted on the Illumina MiSeq platform, Illumina Inc., San Diego, CA, USA [38].

Water quality analysis reagent kits (total nitrogen, total phosphorus, and ammonium nitrogen) were purchased from Lohand Biological. Urease and phosphatase activity assay kits were obtained from Solarbio, Beijing, China. Ultrapure water with a conductivity.

#### 2.3.2. Experimental Design and Operating Conditions

In this study, transparent quartz water tanks with a volume of 5 L (diameter: 15 cm, height: 28 cm) were used and covered with breathable lids. The experiment was initiated on 13 September 2025. Each treatment group (including the unplanted blank control group) was set up with three independent biological replicates (*n* = 3), with one separate tank per replicate. Each tank was filled with 4 L of filtered raw water. Specifically, five individual plants were cultivated in each tank of the planted treatment groups, whereas no plants were introduced in the blank control group. All other culture conditions were kept identical across all groups. The initial fresh weights of plants in each treatment were as follows: *Z*. *latifolia* 62.5 ± 4.0 g, *O*. *javanica* 41.0 ± 2.5 g, *I*. *aquatica* 31.5 ± 2.0 g, *T*. *orientalis* 48.0 ± 3.5 g, *I*. *pseudacorus* 44.5 ± 3.0 g, and *M*. *spicatum* 36.0 ± 2.5 g. The initial biomass of each treatment group was controlled at a similar level to minimize the influence of initial differences on the experimental results.

The experiment was carried out in an artificial climate chamber. The temperature was maintained at 25 ± 1 °C, the light intensity was 300 μmol m^−2^ s^−1^, the photoperiod was 12 h/12 h (light/dark), and the relative humidity was 70% ± 5%. The experimental period lasted for 42 days. Water was replenished daily to maintain a constant volume of 4 L. Sampling was conducted every 7 days starting from day 7. Each time, 100 mL of water samples were collected from the middle layer of the tank for water quality analysis.

### 2.4. Measurement of Indicators

#### 2.4.1. Determination of Water Quality Parameters

Total nitrogen (TN), total phosphorus (TP), and ammonium nitrogen (NH_4_^+^-N) were determined using the alkaline potassium persulfate ultraviolet digestion method (GB 11894-89) [35], ammonium molybdate spectrophotometry (GB 11893-89) [36], and Nessler reagent spectrophotometry (GB 7479-87) [37], respectively. Dissolved oxygen (DO) was measured using the iodometric method, and pH was determined using the glass electrode method.

The pollutant removal rate was calculated using the following equation:

Removal rate (%) = (*C*_0_ − *C_t_*)/*C*_0_ × 100%

where *C*_0_ represents the initial concentration and *C_t_* represents the concentration at time *t*.

#### 2.4.2. Measurement of Plant Growth Parameters

Root length and biomass were measured before and after the experiment. Biomass increase was calculated according to the following equation:

Increase fold = final biomass/initial biomass

Fresh weight was determined using an electronic balance with a precision of 0.01 g.

#### 2.4.3. Determination of Rhizosphere Microorganisms, Enzyme Activities, and Metabolites

At the end of the hydroponic experiment, root-associated samples were collected for microbial, enzyme, and metabolite analyses. Total DNA was extracted using a commercial kit (Vazyme, Nanjing, China). The V3–V4 region of the bacterial 16S rRNA gene was amplified using primers 338F and 806R and sequenced on an Illumina platform. Raw reads were quality-filtered, denoised, merged, and screened for chimeras to obtain amplicon sequence variants (ASVs). Taxonomic annotation was performed against the SILVA 138 database using a Bayesian classifier. Sequencing coverage exceeded 0.999.

Rhizosphere urease and phosphatase activities were determined using commercial kits, with absorbance measured at 578 nm and 405 nm, respectively, and expressed as U g^−1^ h^−1^ (fresh weight basis).

Soluble metabolites were analyzed by HPLC using amino or anion exchange columns with detection at 210 nm.

All microbial, enzyme, and metabolite data were interpreted as descriptive and correlative evidence, without inferring causal functional mechanisms.

### 2.5. Data Analysis

Experimental data were organized, statistically analyzed, and plotted using Microsoft Excel and Origin software. Results are expressed as mean ± standard deviation (mean ± SD). One-way analysis of variance (One-way ANOVA) was used to test the overall differences in the removal efficiency of water quality indicators among different treatment groups. When the differences were significant (*p* < 0.05), Tukey’s multiple comparison test was performed for pairwise comparisons between groups, with the significance level set at *p* < 0.05.

## 3. Results

### 3.1. Growth Characteristics

**Figure 1:**
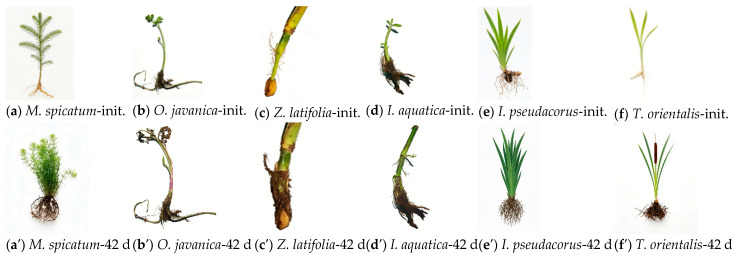
Growth morphology of six wetland plants before and after the 42 d hydroponic experiment.

During the 42 d hydroponic experiment, all six wetland plants grew normally; however, significant differences were observed among species in terms of biomass accumulation and root development characteristics (Figure 1 and Table 2).

During the experimental period, biomass increased significantly in all six plant species, although notable interspecific differences were observed in both growth magnitude and biomass allocation patterns. After 42 days of cultivation, root biomass, shoot biomass, and root length were all markedly higher than their initial values across all species.

Regarding root biomass, the fold increase ranged from 2.3 to 5.1 among the species, indicating clear interspecific variation. The highest increase was observed in *M. spicatum* (5.1-fold), followed by *Z. latifolia* (4.1-fold) and *T. orientalis* (4.0-fold), whereas relatively lower increases were found in *I pseudacorus* and *O javanica* (both 2.3 fold), and *I. aquatica* (2.6-fold). These differences suggest variation in belowground biomass accumulation capacity among species.

In contrast, shoot biomass exhibited greater increases overall, with fold changes ranging from 5.6 to 9.8, consistently exceeding those of root biomass. The highest increases were recorded in *I. aquatica* (9.8-fold) and *O. javanica* (9.2-fold), followed by *T. orientalis* (7.9-fold) and *Z. latifolia* (7.0-fold). *M. spicatum* also showed a relatively rapid increase (6.6-fold), whereas *I. pseudacorus* exhibited the lowest increase (5.6-fold), further highlighting interspecific differences in aboveground growth.

By comparison, root length increased to a lesser extent, with fold changes ranging from 1.9 to 2.6, indicating relatively limited variation. The greatest increase was observed in *I. aquatica* (2.6-fold), followed by *T. orientalis* (2.5-fold) and *M. spicatum* (2.3-fold), while *I. pseudacorus* and *O. javanica* showed smaller increases (1.9-fold), and *Z. latifolia* exhibited a 2.0-fold increase.

In terms of absolute values, *O. javanica* and *I. aquatica* achieved the highest shoot biomass after 42 days (32.21 ± 7.02 and 39.72 ± 8.74, respectively), indicating strong aboveground growth. Meanwhile, *Z. latifolia* exhibited relatively high values in both root and shoot biomass (4.29 ± 0.81 and 35.64 ± 7.21), reflecting robust overall growth performance. Although *M. spicatum* had lower initial biomass, its relatively high fold increase suggested considerable growth potential. Overall, all plant species demonstrated favorable growth during the experimental period. A consistent pattern was observed in which shoot biomass increased more rapidly than root biomass, while root length exhibited comparatively smaller changes. These findings highlight clear interspecific differences in growth strategies and biomass allocation.

### 3.2. Removal Performance of Total Nitrogen (TN)

**Figure 2:**
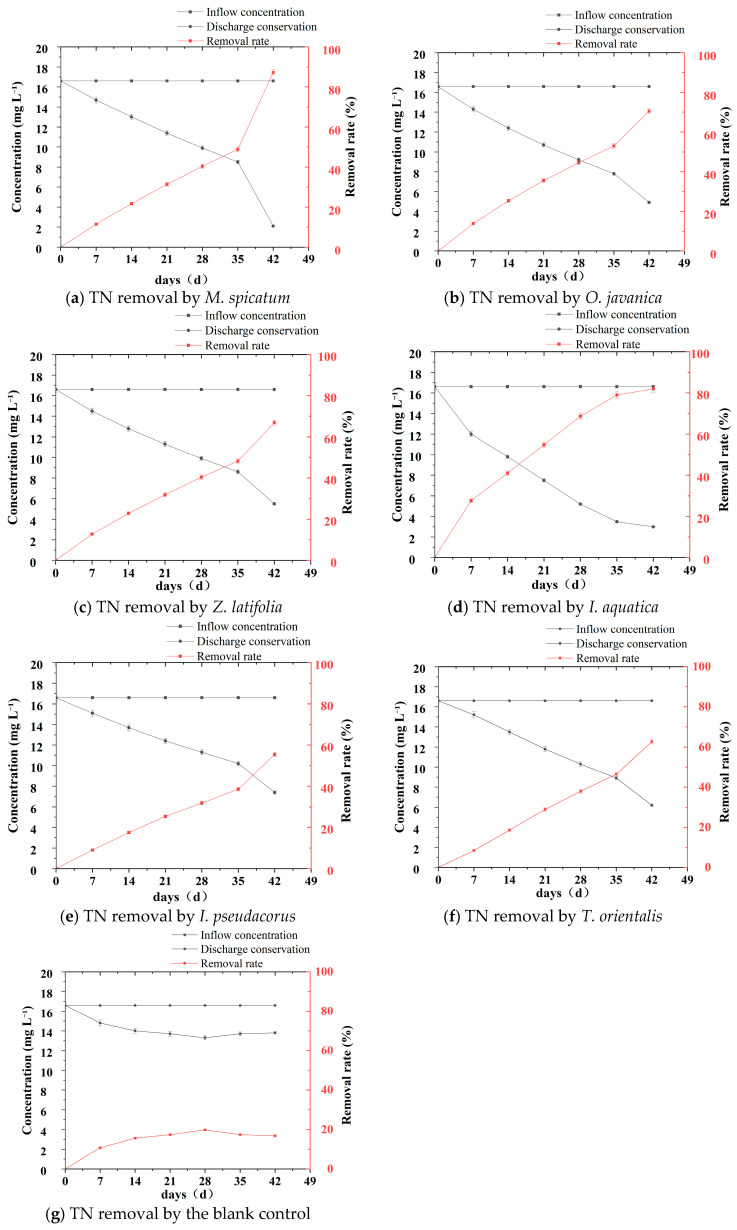
Temporal changes in TN concentration and removal rate for six wetland plants and the blank control during the 42 days hydroponic experiment. (**a**–**g**) TN removal by each plant species and the blank control.

**Figure 3:**
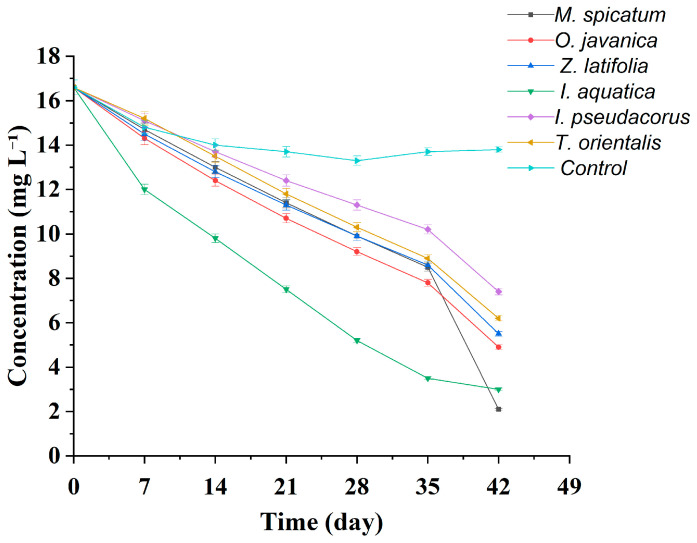
Temporal variations in TN concentration in all wetland plant treatments and the blank control group during the 42 days hydroponic experiment. Note: Values are presented as mean ± standard deviation (*n* = 3).

Significant differences were observed among the wetland plants in their ability to remove TN from eutrophic water. During the experiment, TN concentrations in all plant treatment groups showed a continuous decreasing trend over time (Table 3, Figure 2 and Figure 3), indicating that all six plant species possessed certain nitrogen removal capacities; however, the removal rates and final removal efficiencies differed markedly among the treatments.

According to Table 3 and Figure 3, the TN concentration in the blank control group showed an initial decrease followed by a slight rebound. The initial concentration was 16.6 mg L^−1^, which decreased to 13.3 mg L^−1^ at week 4 and then increased to 13.8 ± 0.14 mg L^−1^ at the end of the experiment (week 6), with a final removal rate of only 16.9%. This trend indicates that the initial decrease in TN concentration was mainly attributable to physical processes such as particulate sedimentation and adsorption onto the container walls. However, in the later stage, the secondary release of nitrogen caused by algal growth and microbial reproduction resulted in a rebound in TN concentration, making it difficult to achieve sustained and efficient nitrogen removal.

In contrast, the *M. spicatum* treatment exhibited the greatest decrease in TN concentration, declining from the initial value of 16.6 mg L^−1^ to 2.1 ± 0.04 mg L^−1^ by week 6, corresponding to a final removal rate of 87.3%, which was significantly higher than those of the other plant treatments. Among the emergent plants, *I*. *aquatica* and *O*. *javanica* showed relatively strong TN removal capacities. At the end of the experiment, the TN concentration in the *I*. *aquatica* treatment decreased to 3.0 ± 0.04 mg L^−1^, corresponding to a removal rate of 81.9%, while the TN concentration in the *O*. *javanica* treatment was 4.9 ± 0.04 mg L^−1^, with a removal rate of 70.5%. The TN concentrations in the *Z*. *latifolia* and *T*. *orientalis* treatments decreased to 5.5 ± 0.05 mg L^−1^ and 6.2 ± 0.05 mg L^−1^, corresponding to removal rates of 66.9% and 62.7%, respectively. In contrast, the *I*. *pseudacorus* treatment showed a relatively smaller decrease in TN concentration, reaching 7.4 ± 0.06 mg L^−1^ at the end of the experiment, with a final removal rate of 55.4%.

From the dynamic variation trends (Figure 2), the TN concentration curves of the *M*. *spicatum* and *I*. *aquatica* treatments remained at relatively low levels throughout the experiment and showed steeper declining slopes. During the first four weeks, TN concentrations decreased from 16.6 mg L^−1^ to 9.9 mg L^−1^ and 8.6 mg L^−1^ in the *M*. *spicatum* and *I*. *aquatica* treatments, respectively, indicating a faster reduction rate than in the other treatments. During weeks 5–6, the TN concentration in the *M*. *spicatum* treatment showed an accelerated decline, dropping sharply from 8.5 mg L^−1^ to 2.1 mg L^−1^. In contrast, the TN concentration curves of treatments such as *I*. *pseudacorus* and *T*. *orientalis* tended to become more gradual without an obvious acceleration stage, further demonstrating the high efficiency and persistence of TN removal by *M*. *spicatum*. Overall, significant differences were observed among the wetland plants in their TN removal capacities. Among them, *M*. *spicatum* exhibited the most prominent removal performance, followed by *I*. *aquatica* and *O*. *javanica*.

### 3.3. Removal Performance of Total Phosphorus (TP)

**Figure 4:**
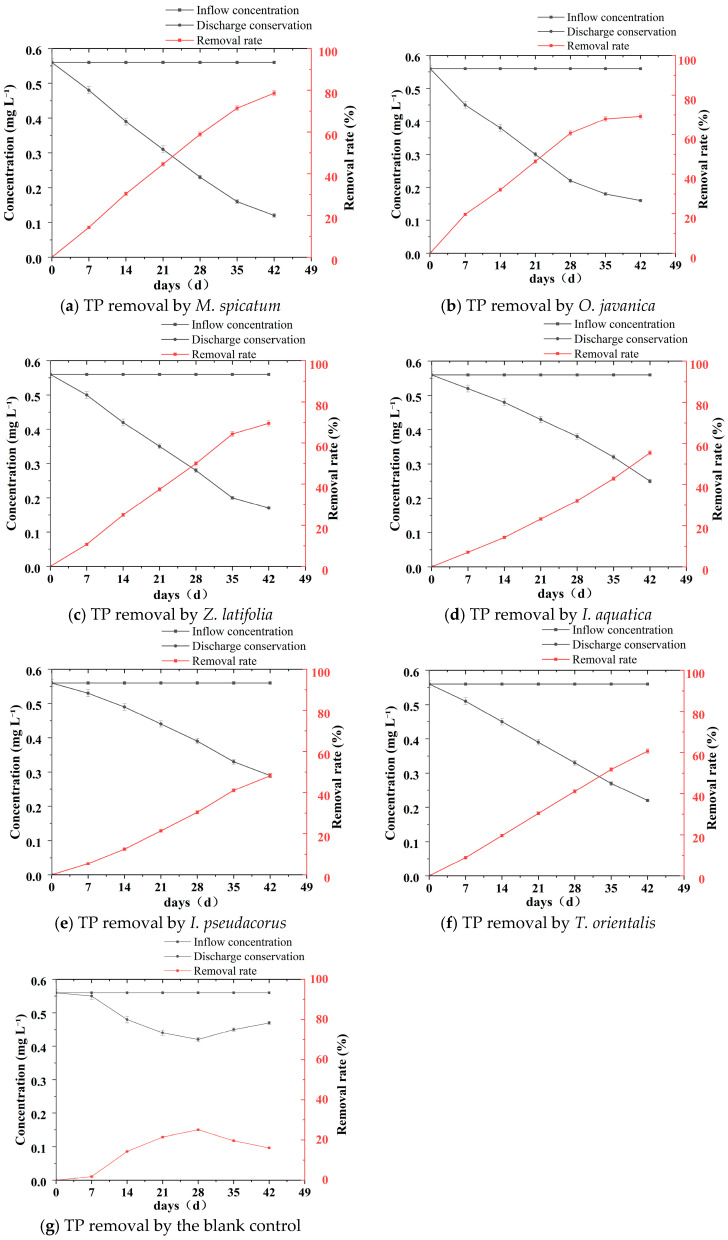
Temporal changes in TP concentration and removal rate for six wetland plants and the blank control during the 42 days hydroponic experiment. (**a**–**g**) TP removal by each plant species and the blank control.

**Figure 5:**
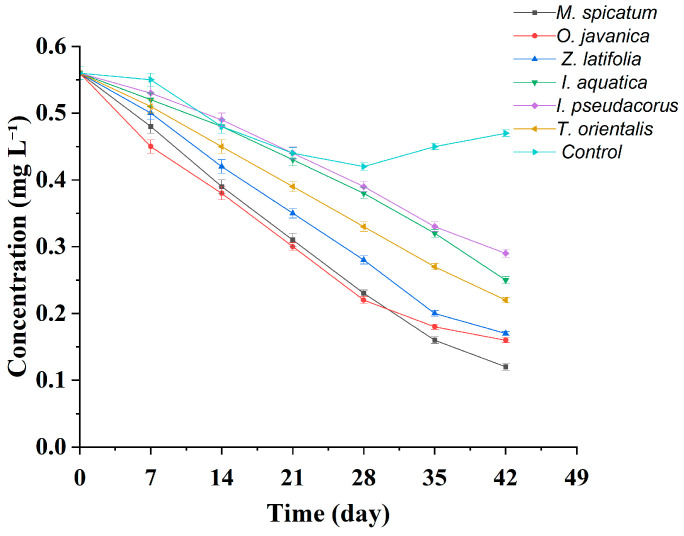
Temporal changes in TP concentration in all wetland plant treatments and the blank control group during the 42 days hydroponic experiment. Note: Values are presented as mean ± standard deviation (*n* = 3).

During the experiment, TP concentrations in all treatment groups showed a continuous decreasing trend over time (Table 4, Figure 4 and Figure 5). However, clear differences were observed among plant species in terms of removal rates and final removal efficiencies.

As shown in Figure 5, the TP concentration in the blank control group showed a continuous decrease during the first three weeks, followed by a gradual increase over the subsequent two weeks. The initial concentration was 0.56 mg L^−1^, which decreased to 0.55 ± 0.01 mg L^−1^ at the first sampling point, mainly due to particulate sedimentation and adsorption onto the container walls. It further declined to 0.44 ± 0.01 mg L^−1^ by week 3. However, during the later stage of the experiment (weeks 4–6), algae and microorganisms proliferated rapidly in the water, and the mineralization of organic phosphorus led to secondary phosphorus release. This resulted in a gradual rebound in TP concentration, which eventually stabilized at 0.47 ± 0.01 mg L^−1^.

In contrast, the TP removal efficiency of all plant treatment groups was significantly higher than that of the blank control group.

As shown in Table 4, the *M*. *spicatum* treatment exhibited the greatest de crease in TP concentration, which gradually declined from the initial value of 0.56 mg L^−1^ to 0.12 ± 0.01 mg L^−1^ in week 6, corresponding to a final removal rate of 78.6%, which was significantly higher than those of the other plant treatments. Among the emergent plants, *Z*. *latifolia* and *O*. *javanica* showed relatively strong TP removal capacities. At the end of the experiment, the TP concentration in the *Z*. *latifolia* treatment was 0.17 ± 0.01 mg L^−1^, corresponding to a removal rate of 69.6%, while that in the *O*. *javanica* treatment was 0.19 ± 0.01 mg L^−1^, with a removal rate of 66.1%. The difference between the two treatments was not significant. The TP concentration in the *T*. *orientalis* treatment was 0.22 ± 0.01 mg L^−1^, corresponding to a removal rate of 60.7%, representing a moderate level among the tested plants. In contrast, the TP removal performance of *I*. *aquatica* and *I*. *pseudacorus* was relatively weaker. At the end of the experiment, the TP concentration in the *I*. *aquatica* treatment was 0.25 ± 0.01 mg L^−1^, corresponding to a removal rate of 55.4%, while that in the *I*. *pseudacorus* treatment was 0.29 ± 0.01 mg L^−1^, with a removal rate of 48.2%. The TP removal efficiencies of the *M*. *spicatum*, *Z*. *latifolia*, and *O*. *javanica* treatments were significantly higher than those of the *I*. *aquatica*, *T*. *orientalis*, and *I*. *pseudacorus* treatments. From the dynamic variation trends (Figure 3), the TP concentration curve of the *M*. *spicatum* treatment remained at the lowest level throughout the experiment and showed the steepest declining slope. During the first four weeks, TP concentration decreased from 0.56 mg L^−1^ to 0.23 mg L^−1^ and continued to decline steadily during the later stages without obvious fluctuations. In contrast, the curves of the *I*. *pseudacorus* and *I*. *aquatica* treatments decreased more gradually, and a slight stagnation was even observed during weeks 5–6, further confirming their relatively lower TP removal efficiencies.

### 3.4. Removal Performance of Ammonium Nitrogen (NH_4_^+^-N)

**Figure 6:**
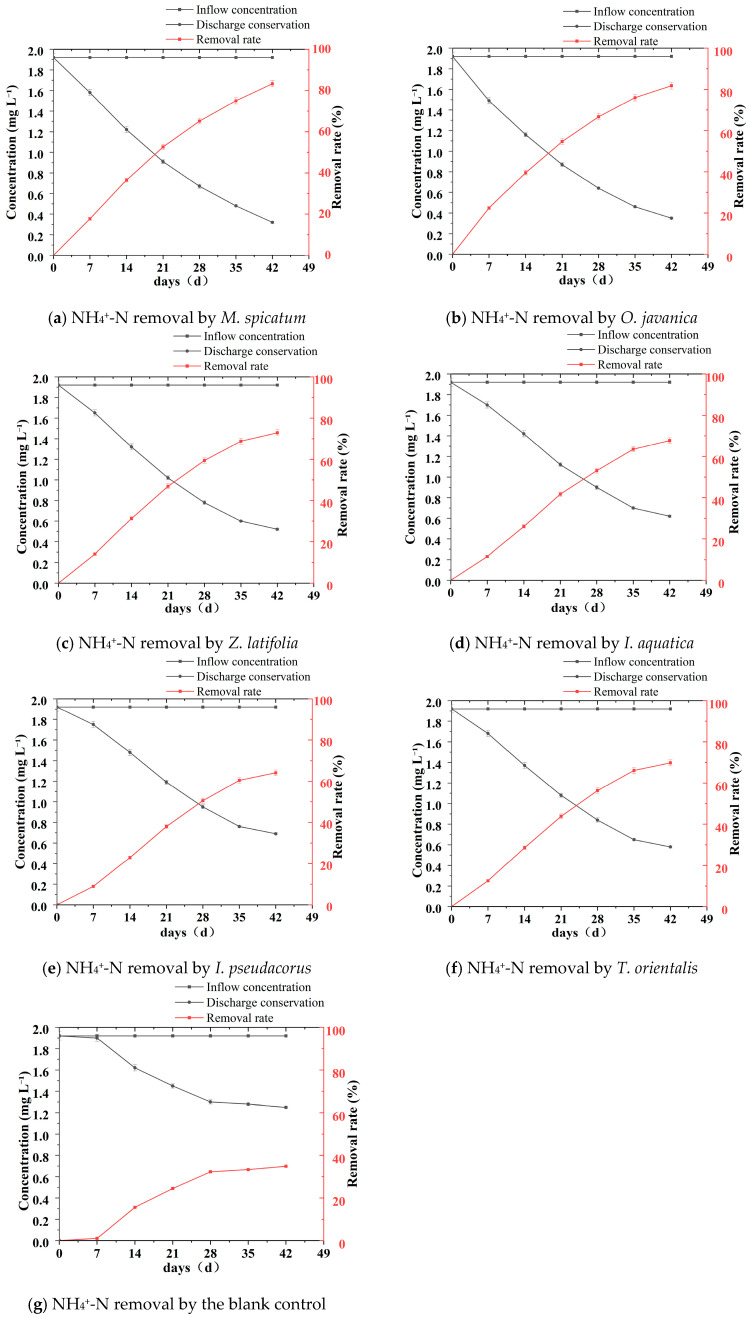
Temporal changes in NH_4_^+^-N concentration and removal rate for six wetland plants and the blank control during the 42 days hydroponic experiment. (**a**–**g**) NH_4_^+^-N removal by each plant species and the blank control.

**Figure 7:**
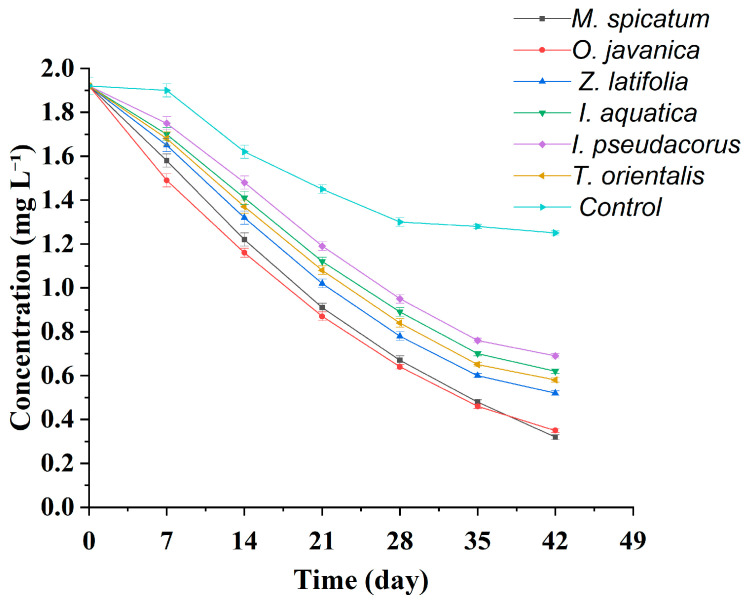
Temporal changes in ammonia nitrogen (NH_4_^+^-N) concentration in all wetland plant treatments and the blank control group during the 42 days hydroponic experiment. Note: Values are presented as mean ± standard deviation (*n* = 3).

During the experiment, the NH_4_^+^-N concentrations in all treatment groups showed a continuous decreasing trend with cultivation time (Table 5, Figure 6 and Figure 7). Overall, the decline was relatively rapid during the first 3–4 weeks and gradually slowed in the later stages. However, clear differences were observed among plant species in terms of removal rates and final removal efficiencies.

As shown in Figure 7, the ammonium nitrogen concentration in the control group showed a gradual and continuous decline followed by stabilization. The initial concentration was 1.92 mg L^−1^, which decreased to 1.25 ± 0.02 mg L^−1^ at the end of the experiment (week 6), with a final removal rate of only 34.9%. This suggests that the reduction in ammonium nitrogen was mainly attributable to processes such as ammonia volatilization and microbial nitrification, resulting in limited removal efficiency. In comparison, all plant treatment groups exhibited significantly higher ammonium nitrogen removal efficiencies than the control group.

As shown in Table 5, by week 6 the NH_4_^+^-N concentration in the *M*. *spicatum* treatment decreased to 0.32 ± 0.01 mg L^−1^, corresponding to a removal rate of 83.3%, which was significantly higher than those of the other plant treatments. The NH_4_^+^-N concentration in the *O*. *javanica* treatment was 0.35 ± 0.01 mg L^−1^, corresponding to a removal rate of 81.8%, and the difference between this treatment and the *M*. *spicatum* treatment was not significant. The NH_4_^+^-N concentrations in the *Z*. *latifolia* and *T*. *orientalis* treatments decreased to 0.52 ± 0.01 mg L^−1^ and 0.58 ± 0.01 mg L^−1^, corresponding to removal rates of 72.9% and 69.8%, respectively. In contrast, the *I*. *aquatica* and *I*. *pseudacorus* treatments exhibited relatively lower removal performance. At the end of the experiment, the NH_4_^+^-N concentrations in these two treatments were 0.62 ± 0.01 mg L^−1^ and 0.69 ± 0.01 mg L^−1^, corresponding to removal rates of 67.7% and 64.1%, respectively.

From the dynamic variation trends (Figure 6), the NH_4_^+^-N concentration curves of the *M*. *spicatum* and *O*. *javanica* treatments remained at the lowest levels throughout the experiment and showed steeper declining slopes, indicating faster ammonium removal rates. In contrast, the curves of the *I*. *pseudacorus* and *I*. *aquatica* treatments declined more gradually, and slight fluctuations even appeared in the later stages, further confirming their relatively lower removal efficiencies. The NH_4_^+^-N removal efficiencies of the *M. spicatum* and *O. javanica* treatments were significantly higher than those of the other four plant treatments. The removal efficiencies of the *Z. latifolia* and *T. orientalis* treatments were significantly higher than those of the *I. aquatica* and *I. pseudacorus* treatments, while no obvious difference was observed between the latter two treatments.

### 3.5. Empirical Fitting of Nutrient Reduction Trends

**Figure 8:**
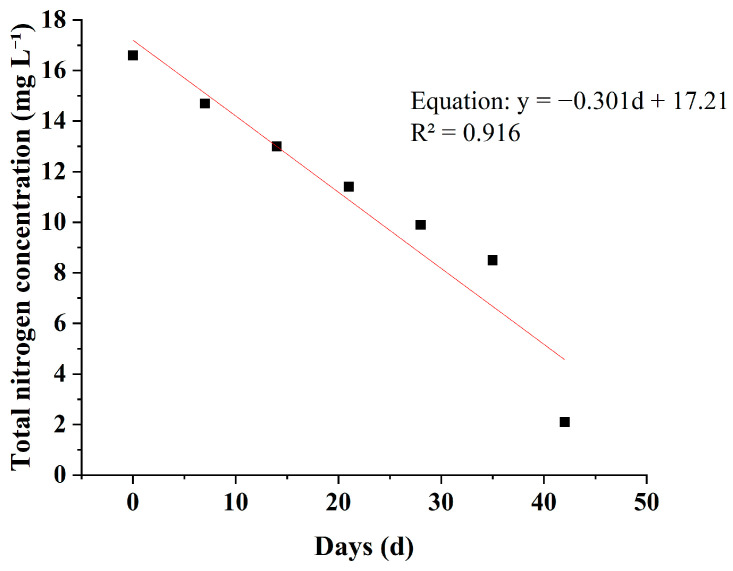
Empirical linear fit of TN concentration over time in the *M. spicatum* treatment.

**Figure 9:**
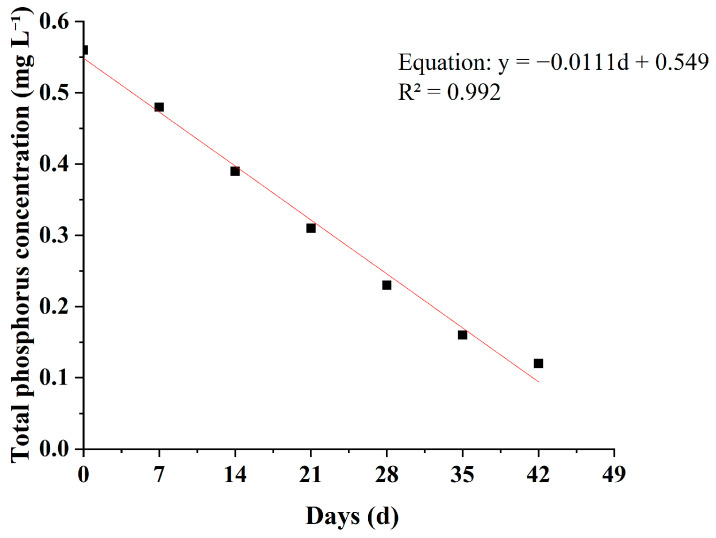
Empirical linear fit of TP concentration over time in the *M. spicatum* treatment.

**Figure 10:**
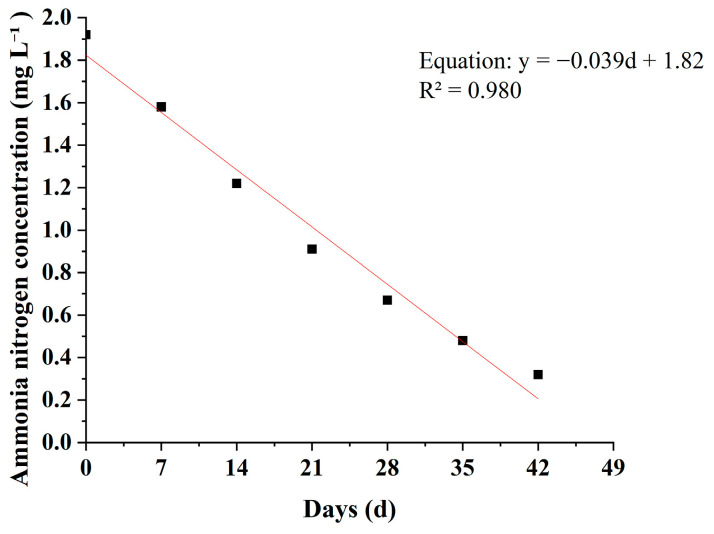
Empirical linear fit of NH_4_^+^-N concentration over time in the *M. spicatum* treatment.

To analyze the kinetic characteristics of pollutant removal, linear regression analysis was performed on the relationships between TN, TP, and NH_4_^+^-N concentrations and cultivation time in the *M*. *spicatum* treatment, which showed the best removal performance (Figure 8, Figure 9 and Figure 10). The results showed that the concentrations of the three pollutants were significantly negatively correlated with cultivation time. The fitted equations were as follows:

TN: y = −0.301d + 17.21 (R^2^ = 0.916),

TP: y = −0.011d + 0.549 (R^2^ = 0.992),

NH_4_^+^-N: y = −0.039d + 1.82 (R^2^ = 0.980).

In these equations, y represents the pollutant concentration (mg L^−1^), and d represents the cultivation time (d). The coefficients of determination (R^2^) for all models were greater than 0.91, indicating a strong linear correlation between pollutant concentration and time during the experimental period.

It should be noted that, under real environmental conditions, nutrient removal processes in plant-based treatment systems may also exhibit nonlinear behavior rather than being predominantly linear. The linear regression applied in this study serves only as an empirical description of the temporal trends observed during the experimental period. Future studies should focus on comparing and optimizing more representative kinetic models (e.g., first-order and second-order kinetics), rather than relying solely on linear fitting.

Compared with TN (R^2^ = 0.916) and NH_4_^+^-N (R^2^ = 0.980), the fitting accuracy for TP (R^2^ = 0.992) was higher, suggesting that the TP removal process by *M. spicatum* exhibited a more pronounced linear relationship with time.

### 3.6. Rhizosphere Microbial Community Characteristics

#### 3.6.1. Community Composition Characteristics

**Figure 11:**
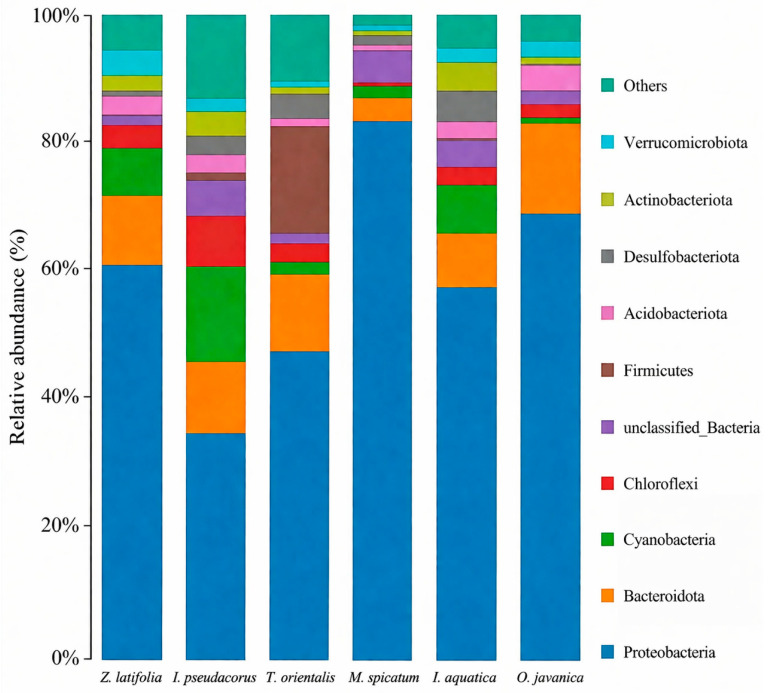
Composition and distribution of root-associated microbial communities at the phylum level for six wetland plants.

High-throughput sequencing revealed clear differences in root-associated microbial communities among the six wetland plant species (Figure 11). At the phylum level, *Proteobacteria* was the dominant taxon across all treatments. Notably, the relative abundance of *Proteobacteria* was highest in the *M. spicatum*-associated community, exceeding 80%. In addition to *Proteobacteria*, other bacterial phyla, including *Actinobacteriota*, *Bacteroidota*, and *Firmicutes*, were also detected; however, their relative abundances were lower and varied among plant species.

#### 3.6.2. Distribution of Taxonomically Inferred Functional Groups

**Figure 12:**
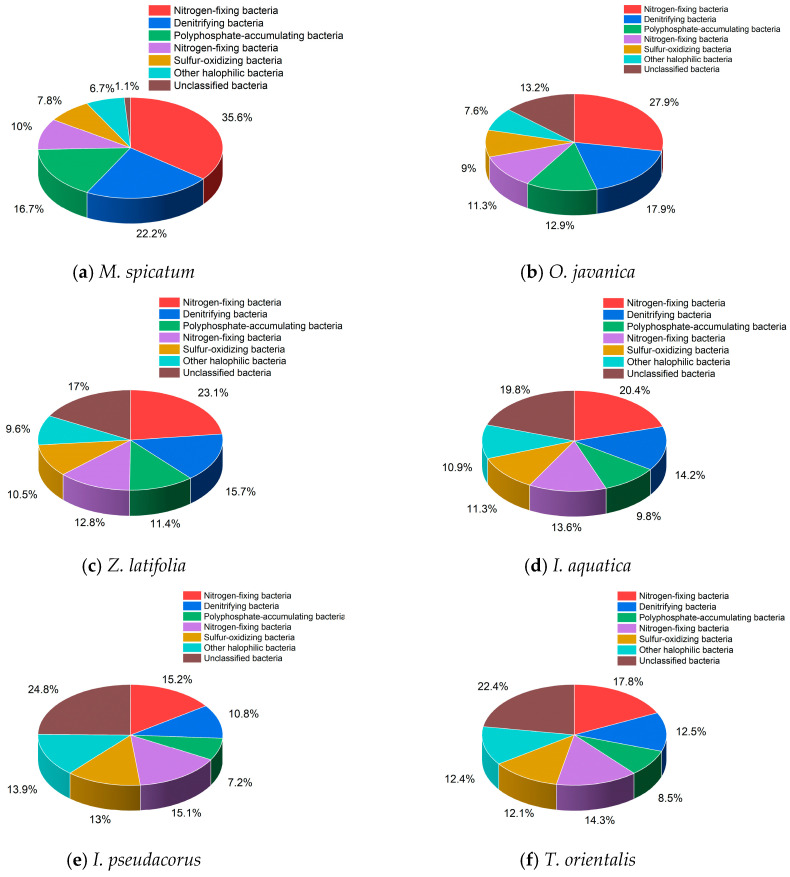
Relative abundance of taxonomically inferred nitrogen- and phosphorus-cycling bacterial groups in the rhizosphere of six wetland plants. Note: The data were derived from 16S rRNA high-throughput sequencing, and only taxonomically inferred major groups within Proteobacteria are presented.

Analysis of *Proteobacteria*-associated taxa related to nitrogen and phosphorus cycling (Figure 12) showed clear differences in their relative abundances among plant rhizospheres. The rhizosphere of *M. spicatum* exhibited higher proportions of taxa assigned to putative *nitrogen-fixing bacteria*, *denitrifying bacteria*, and *polyphosphate-accumulating organisms* (PAOs) (35.6%, 22.2%, and 16.7%, respectively) compared with the other treatments. *O. javanica* and *Z. latifolia* showed intermediate levels, whereas *I. pseudacorus* and *I. aquatica* exhibited relatively lower proportions. Distinct differences in microbial community composition were observed among the plant treatments.

The distribution patterns of these taxa were generally consistent with the nutrient removal performance of the plants, consistent with previous reports [39,40]. Specifically, *M. spicatum* showed the highest efficiency, followed by *O. javanica* and *Z. latifolia*, whereas *I. pseudacorus* and *I. aquatica* exhibited lower performance. It should be noted that these results are based on 16S rRNA gene sequencing and taxonomic annotation, and the functional roles described here are putative.

#### 3.6.3. Alpha Diversity Analysis

The alpha diversity indices of rhizosphere microbial communities (Table 6) showed that the sequencing coverage of all samples exceeded 0.999, indicating that the sequencing depth was sufficient for community composition analysis. In terms of species richness (ACE and Chao1), *T. orientalis* exhibited the highest values, followed by *O. javanica* and *I. pseudacorus*, whereas *I. aquatica* showed the lowest richness. The Shannon index was highest for *T. orientalis* (10.1091) and *I. pseudacorus* (9.9596), intermediate for *O. javanica* and *Z. latifolia*, and lowest for *I. aquatica* (5.7499). Phylogenetic diversity (PD_whole_tree) was relatively high in *I. pseudacorus*, *Z. latifolia*, and *T. orientalis*. These results indicate that community richness and diversity did not correspond to pollutant removal performance in a simple linear manner.

## 4. Discussion

### 4.1. Coupling Relationship Between Biomass Accumulation and Nutrient Removal

It was demonstrated in this study that the biomass accumulation rates of different wetland plants were generally positively correlated with their capacities for nitrogen and phosphorus removal. During the experimental period, greater increases in both root and shoot biomass were observed in *M. spicatum* and *O*. *javanica*, which were concurrently associated with higher removal efficiencies of total nitrogen (TN), total phosphorus (TP), and NH_4_^+^-N. These findings suggested that plant assimilation contributed substantially to the remediation of eutrophic water bodies.

It was observed that the submerged *M. spicatum* exhibited rapid biomass accumulation. Meanwhile, its fully submerged shoot system maintained extensive contact with the surrounding water, thereby enlarging the nutrient uptake interface and enhancing the assimilation capacity for inorganic nitrogen and phosphorus. Consequently, the increased biomass also functioned as a larger biological sink for nutrients, providing a robust material basis for the sustained removal of pollutants.

However, biomass alone was not the sole determinant of removal efficiency. For example, although the biomass growth rate of *O*. *javanica* was comparable to that of *M*. *spicatum*, its TN and TP removal efficiencies were still lower. This difference may be attributed to the structural characteristics of submerged plants. As a submerged species, *M*. *spicatum* can directly absorb nutrients through its submerged stems and leaves, while these structures also provide extensive surfaces for microbial colonization, forming a “plant–microbe” composite purification system. In contrast, emergent plants mainly interact with water through their root systems, resulting in smaller interfaces for nutrient uptake and microbial attachment. This finding further supports the structural advantages of submerged plants in nutrient removal processes.

### 4.2. Root Structure and Plant–Microbe Synergistic Mechanisms

Differences in rhizosphere microbial community structure were associated with variations in purification efficiency among different plant species. High-throughput sequencing results showed that *Proteobacteria* was the dominant bacterial phylum in the rhizospheres of all plants, with a relatively higher abundance observed in *M. spicatum* compared to the other species. Members of *Proteobacteria* are widely reported to include taxa involved in nitrogen and phosphorus cycling processes, such as nitrification, denitrification, and phosphorus accumulation. Accordingly, the higher relative abundance of taxa classified as putative nitrifying bacteria, denitrifying bacteria, and polyphosphate-accumulating organisms (PAOs) in the rhizosphere of *M. spicatum* may be associated with enhanced nitrogen transformation and phosphorus retention, potentially contributing to its superior purification performance.

Notably, the rhizosphere microbial community of *M. spicatum* exhibited relatively lower alpha diversity but a higher proportion of taxa associated with nitrogen and phosphorus cycling. This pattern may indicate a degree of functional specialization within its microbial community structure [41], which could favor specific metabolic pathways related to nutrient removal. However, it should be emphasized that these functional interpretations are based on 16S rRNA gene sequencing and taxonomic annotation, and therefore remain putative. The absence of metagenomic or functional assays limits the ability to directly link microbial composition with metabolic activity or process rates. Future studies incorporating metagenomic approaches and functional analyses are needed to validate these potential functional roles and to better elucidate the underlying mechanisms. In contrast, the rhizosphere microbial communities of *O. javanica* and *Z. latifolia* exhibited higher diversity and a more balanced composition, which may confer greater resilience to environmental fluctuations.

These results suggest that microbial communities with a higher relative abundance of taxa associated with nitrogen and phosphorus cycling may, to some extent, compensate for lower microbial diversity and contribute to enhanced nutrient removal efficiency.

### 4.3. Mechanistic Analysis of Multi-Factor Coupling Effects

The superior purification performance of *M. spicatum* resulted from the combined effects of biomass accumulation, morphological characteristics, and rhizosphere microbial functions. First, rapid biomass accumulation enhanced the direct assimilation of nitrogen and phosphorus by plants, forming a stable biological nutrient sink. Second, the morphological characteristics of submerged plants allowed their stems and leaves to maintain extensive contact with the surrounding water, which not only increased the interface for nutrient exchange but also provided abundant attachment sites for microorganisms. Third, the high abundance of *Proteobacteria* and other functional microbial groups involved in nitrogen and phosphorus cycling in the rhizosphere further removed residual nutrients through microbial transformations, such as nitrification–denitrification and biological phosphorus accumulation. These processes together formed a synergistic purification pathway involving “plant uptake + microbial transformation”.

This bidirectional interaction between plants and microorganisms not only ensured continuous pollutant removal but also enhanced the operational stability of the system. It therefore represents the key mechanism underlying the superior nitrogen and phosphorus removal performance of *M. spicatum*, which is consistent with current findings in wetland ecological restoration regarding plant–microbe synergistic processes.

### 4.4. Implications for Ecological Restoration Practices

The results of this study provide important experimental evidence for plant selection and ecological restoration strategies in eutrophic water bodies. Submerged plants generally exhibit greater potential for nutrient removal under conditions of low turbidity and low flow velocity, whereas emergent plants possess advantages in environmental adaptability, structural stability, and ease of management in engineering applications. In practical ecological restoration projects, the construction of multi-layered purification systems through the complementary integration of submerged and emergent plants can enhance the resistance of restoration systems to environmental disturbances and improve long-term operational stability.

These findings are generally consistent with previous studies [14,42], which have reported that submerged macrophytes such as *Hydrilla verticillata* and *Vallisneria natans* play a dominant role in nutrient uptake and water clarity improvement under stable conditions. In contrast, emergent species such as *Phragmites australis* and *Typha latifolia* are more resilient to fluctuating hydrological regimes and are widely used in constructed wetlands due to their robustness and ease of maintenance. However, some studies have suggested that the efficiency of submerged plants may decline significantly under conditions of high turbidity or disturbance, highlighting the importance of integrating different functional plant groups to achieve more stable restoration outcomes. Compared with these studies, the present results further emphasize the synergistic effects of plant combinations under controlled conditions, providing additional experimental support for integrated vegetation design in eutrophic water restoration.

Compared with existing comparative studies, the present work provides novel insights by systematically evaluating six wetland plant species under identical raw water and hydroponic conditions. By simultaneously linking nutrient removal performance, biomass accumulation, and rhizosphere microbial community structure within a unified experimental framework, this study further elucidates the coupling mechanisms underlying interspecific differences in purification efficiency. These findings provide new empirical evidence and a more comprehensive comparative framework for optimizing integrated vegetation configuration in the practical restoration of eutrophic water bodies.

However, this study was conducted under indoor hydroponic conditions, and the practical applicability of the findings still requires further validation under natural environmental conditions. Future research should therefore include in situ field experiments in natural water bodies, such as the Qilu Lake basin, integrating hydrological and water quality characteristics to optimize plant configuration strategies for watershed-scale restoration.

## 5. Conclusions

During the 42-day hydroponic experiment, all six wetland plant species reduced the concentrations of TN, TP, and NH_4_^+^-N in eutrophic water, although substantial differences in purification performance were observed among species. Among the tested species, *M. spicatum* exhibited the highest removal efficiencies for all three nutrient indicators and maintained relatively stable removal trends over time. The results further indicate that greater biomass accumulation is generally associated with enhanced nutrient removal, suggesting that plant growth and nutrient assimilation play important roles in pollutant reduction within the experimental systems. In addition, the *M. spicatum* treatment exhibited a distinct microbial community pattern, characterized by a relatively higher abundance of microbial groups associated with nitrogen and phosphorus cycling. This pattern may have contributed to the superior purification performance observed in this treatment.

However, the present study demonstrates an association rather than a confirmed causal relationship, and microbial community composition should therefore be regarded as a potentially important influencing factor rather than a definitively established key driver. Overall, the relatively high purification capacity of *M. spicatum* under the tested conditions is likely attributable to the combined effects of plant growth characteristics, submerged morphology, and plant-associated microbial functions.

Nevertheless, as this study was conducted in a simplified indoor hydroponic system lacking sediment–water interactions, hydrodynamic processes, seasonal variation, and other sources of environmental complexity, the results should be extrapolated to natural aquatic ecosystems with caution. Further mesocosm and field-based studies are required to validate these findings and to assess the practical applicability of plant selection and configuration strategies for eutrophic water restoration.

## Figures and Tables

**Table 1:** Physicochemical characteristics of the raw water used in the experiment.

Water Quality Parameter	TN (mg L^−1^)	TP (mg L^−1^)	NH_4_^+^-N (mg L^−1^)	pH	DO (mg L^−1^)
Initial concentration	16.6 ± 0.30	0.56 ± 0.02	1.92 ± 0.05	7.8 ± 0.2	5.5 ± 0.3
Determination method	Alkaline potassium persulfate-UV spectrophotometry [35]	Ammonium molybdate spectrophotometry [36]	Nessler reagent spectrophotometry [37]	Glass electrode method	Iodometric method

Note: “Initial concentration” shows the mean ± SD initial levels of TN, TP, NH_4_^+^-N, pH, and DO. All determinations were performed following the specified Chinese national standard methods (GB).

**Table 2:** Growth Parameters and Biomass Increase in Six Plant Species (Length: cm; Mass: g).

Plant Species	Initial Biomass			Biomass at Day 42			Biomass Increase (Fold)		
	Root	Shoot	Root Length	Root	Shoot	Root Length	Root	Shoot	Root Length
*M. spicatum*	0.32 ± 0.04	2.04 ± 0.21	3.2 ± 0.4	1.63 ± 0.28	13.39 ± 2.46	7.4 ± 0.9	5.1	6.6	2.3
*O. javanica*	1.32 ± 0.19	3.52 ± 0.74	7.7 ± 1.2	2.98 ± 0.46	32.21 ± 7.02	14.8 ± 2.6	2.3	9.2	1.9
*Z. latifolia*	1.04 ± 0.17	5.07 ± 0.91	6.2 ± 0.9	4.29 ± 0.81	35.64 ± 7.21	12.2 ± 2.4	4.1	7.0	2.0
*I. aquatica*	1.25 ± 0.11	4.07 ± 0.84	5.6 ± 0.8	3.27 ± 0.70	39.72 ± 8.74	14.5 ± 3.7	2.6	9.8	2.6
*I. pseudacorus*	0.86 ± 0.12	4.24 ± 0.88	4.3 ± 0.6	1.96 ± 0.44	23.66 ± 4.53	8.1 ± 1.5	2.3	5.6	1.9
*T. orientalis*	0.44 ± 0.08	2.13 ± 0.27	3.8 ± 0.6	1.78 ± 0.38	16.87 ± 3.82	9.5 ± 1.8	4.0	7.9	2.5

**Table 3:** Changes in TN concentration during the 42 days hydroponic experiment for six wetland plants (mg L^−1^).

Sampling Time (Week)	*M. spicatum*	*O. javanica*	*Z. latifolia*	*I. aquatica*	*I. pseudacorus*	*T. orientalis*	Ctrl (No Plants)
1	14.7 ± 0.16	14.3 ± 0.14	14.5 ± 0.14	14.0 ± 0.12	15.1 ± 0.17	15.0 ± 0.17	14.8 ± 0.15
2	13.0 ± 0.14	12.4 ± 0.11	12.8 ± 0.12	12.0 ± 0.10	13.7 ± 0.15	13.6 ± 0.15	14.0 ± 0.14
3	11.4 ± 0.12	10.7 ± 0.09	11.3 ± 0.10	10.2 ± 0.08	12.4 ± 0.13	12.3 ± 0.13	13.7 ± 0.13
4	9.9 ± 0.10	9.2 ± 0.08	9.9 ± 0.09	8.6 ± 0.07	11.3 ± 0.12	11.1 ± 0.12	13.3 ± 0.12
5	8.5 ± 0.08	7.8 ± 0.06	8.6 ± 0.08	7.2 ± 0.06	10.2 ± 0.10	10.1 ± 0.10	13.7 ± 0.13
6	2.1 ± 0.04	4.9 ± 0.04	5.5 ± 0.05	3.0 ± 0.04	7.4 ± 0.06	6.2 ± 0.05	13.8 ± 0.14

**Table 4:** Changes in TP concentration during the 42 days hydroponic experiment for six wetland plants (mg L^−1^).

Sampling Time (Week)	*M. spicatum*	*O. javanica*	*Z. latifolia*	*I. aquatica*	*I. pseudacorus*	*T. orientalis*	Ctrl (No Plants)
1	0.48 ± 0.01	0.45 ± 0.01	0.50 ± 0.01	0.52 ± 0.01	0.53 ± 0.01	0.51 ± 0.01	0.55 ± 0.01
2	0.39 ± 0.01	0.38 ± 0.01	0.42 ± 0.01	0.48 ± 0.01	0.49 ± 0.01	0.45 ± 0.01	0.48 ± 0.01
3	0.31 ± 0.01	0.30 ± 0.01	0.35 ± 0.01	0.43 ± 0.01	0.44 ± 0.01	0.39 ± 0.01	0.44 ± 0.01
4	0.23 ± 0.01	0.22 ± 0.01	0.28 ± 0.01	0.38 ± 0.01	0.39 ± 0.01	0.33 ± 0.01	0.42 ± 0.01
5	0.16 ± 0.01	0.18 ± 0.01	0.20 ± 0.01	0.32 ± 0.01	0.33 ± 0.01	0.27 ± 0.01	0.45 ± 0.01
6	0.12 ± 0.01	0.19 ± 0.01	0.17 ± 0.01	0.25 ± 0.01	0.29 ± 0.01	0.22 ± 0.01	0.47 ± 0.01

**Table 5:** Changes in NH_4_^+^-N concentration during the 42 days hydroponic experiment for six wetland plants (mg L^−1^).

Sampling Time (Week)	*M. spicatum*	*O. javanica*	*Z. latifolia*	*I. aquatica*	*I. pseudacorus*	*T. orientalis*	Ctrl (No Plants)
1	1.58 ± 0.03	1.49 ± 0.03	1.65 ± 0.03	1.70 ± 0.03	1.75 ± 0.03	1.68 ± 0.03	1.90 ± 0.03
2	1.22 ± 0.02	1.16 ± 0.02	1.32 ± 0.02	1.41 ± 0.02	1.48 ± 0.02	1.37 ± 0.02	1.62 ± 0.02
3	0.91 ± 0.02	0.87 ± 0.02	1.02 ± 0.02	1.12 ± 0.02	1.19 ± 0.02	1.08 ± 0.02	1.45 ± 0.02
4	0.67 ± 0.01	0.64 ± 0.01	0.78 ± 0.01	0.89 ± 0.01	0.95 ± 0.01	0.84 ± 0.01	1.30 ± 0.02
5	0.48 ± 0.01	0.46 ± 0.01	0.60 ± 0.01	0.70 ± 0.01	0.76 ± 0.01	0.65 ± 0.01	1.28 ± 0.02
6	0.32 ± 0.01	0.35 ± 0.01	0.52 ± 0.01	0.62 ± 0.01	0.69 ± 0.01	0.58 ± 0.01	1.25 ± 0.02

**Table 6:** Alpha diversity indices of rhizosphere microbial communities for six wetland plants.

Plant Species	Feature	ACE	Chao1	Simpson	Shannon	PD_Whole_Tree	Coverage
***M. spicatum***	2647	2651.7	2647.0	0.8948	7.7459	20.3066	0.9997
***O. javanica***	3625	3631.2	3625.1	0.9471	8.4157	23.942	0.9997
***Z. latifolia***	2637	2643.2	2637.1	0.9793	8.3166	28.3661	0.9997
***I. aquatica***	1984	1992.8	1984.3	0.8896	5.7499	21.5422	0.9996
***I. pseudacorus***	3458	3466.6	3458.2	0.9966	9.9596	29.0383	0.9996
***T. orientalis***	4319	4326.2	4319.1	0.9963	10.1091	26.231	0.9996

## Data Availability

The original contributions presented in this study are included in the article. Further inquiries can be directed to the corresponding author.
